# Physical activity and factors associated with the costs of low back pain among adults after 18 months of follow-up: a cohort study

**DOI:** 10.1590/1516-3180.2023.0343.R1.03072024

**Published:** 2025-03-17

**Authors:** Everton Alex Carvalho Zanuto, Valter Penna, Cristiano Rocha da Silva, Enio Ricardo Vaz Ronque, Ruben de Faria Negrão, Robson Chacon Castoldi, Jamile Sanches Codogno, Rômulo Araújo Fernandes

**Affiliations:** I Professor, Department of Physical Education, Universidade do Oeste Paulista (UNOESTE), Presidente Prudente (SP), Brazil; Research, Postgraduate Program in Movement Sciences, Universidade Estadual Paulista (UNESP), Presidente Prudente (SP), Brazil.; II Faculdade de Medicina de São José do Rio Preto (FAMERP), São José do Rio Preto (SP), Brazil.; III Researcher, Biomedical Engineering Laboratory, Escola Politécnica, Universidade de São Paulo (USP), São Paulo (SP), Brazil.; IV Professor, Department of Physical Education, Universidade Estadual de Londrina (UEL), (PR), Brazil.; V Professor, Postgraduate Program in Movement Sciences, Department of Physiotherapy, Universidade Estadual Paulista (UNESP), Presidente Prudente (SP), Brazil.; VI Researcher, Postgraduate Program in Movement Sciences, Universidade Estadual Paulista (UNESP), Rio Claro (SP), Brazil; Professor of the Graduate Program in Physical Exercise in Health Promotion. Universidade Norte do Paraná (UNOPAR), Londrina (PR), Brazil.; VII Professor, Postgraduate Program in Movement Sciences, Universidade Estadual Paulista (UNESP), Presidente Prudente (SP), Brazil.; VIII Professor, Postgraduate Program in Movement Sciences, Universidade Estadual Paulista (UNESP), Presidente Prudente (SP), Brazil.

**Keywords:** Public health administration, Body composition, Economics, Prevention and control [subheding], Exercise, Costs control, Costs of health, Physical exercise, Backaches, Surveys and Questionnaires

## Abstract

**BACKGROUND:**

Chronic low back pain (CLBP) is a substantial health problem that causes considerable economic losses. Several studies have demonstrated the protective effect of habitual physical activity; however, little data are available regarding its impact on the costs associated with CLBP.

**OBJECTIVES:**

The primary aim of this study was to analyze the costs of CLBP in the Brazilian Health System and associated factors among adults.

**DESIGN AND SETTING:**

An 18-month cohort study was conducted in two basic health units in Presidente Prudente (SP), Brazil.

**METHODS:**

A total of 198 patients were interviewed and evaluated four times: at baseline, with retrospective data covering the previous 12 months, and at six, 12, and 18 months. The Nordic and Baecke questionnaires were used to classify CLBP, and the Baecke questionnaire was used for physical activity assessment. The costs were calculated by reviewing the demand for services from medical records. Body mass index (kg/m^2^) was determined using body mass and height values collected during the interviews. The questionnaire included confounding variables, such as sex, age, ethnicity, and socioeconomic status.

**RESULTS:**

A high prevalence of CLBP was observed, which was associated with female sex and younger age. CLBP resulted in high costs for medical consultations (without: US$ 34.25 ± 23.21; with: US$ 39.62 ± 27.25; P = 0.049), while cycling was negatively associated with costs (rho = -0.289; P = 0.049).

**CONCLUSION:**

Lower back pain was associated with higher costs of medical consultations, while cycling was associated with reduced costs.

## INTRODUCTION

The epidemiological aspects of chronic low back pain (CLBP) have been analyzed worldwide, primarily because CLBP is highly prevalent among adults.^1,2^ In developing nations, the prevalence of CLBP ranges from 4.2% to 14.7%, while any episode of CLBP during a given year affects 50–60% of the population.^3,4^

According to Barrey et al.,^5^ CLBP is a serious public health problem, and spending on its treatment has steadily increased over the last four decades. This financial burden can be attributed to various therapeutic interventions, such as medical consultations, physiotherapy sessions, imaging tests, and medications.^5^

CLBP is a substantial health issue, ranking as the second most common cause of medical consultations worldwide.^6^ In developed countries, such as the United States, United Kingdom, and Australia,^7,8,9,10^ CLBP leads to considerable economic losses; however, little is known about its economic burden in developing settings.

Furthermore, CLBP is a major cause of work absence. In addition to financial costs, CLBP has psychological and social consequences for patients, reducing productivity and limiting mobility, which may hinder socialization.^2,11,12^

Studies have demonstrated the protective effect of habitual physical activity (PA), distinct from exercise protocols conducted in laboratory settings, on various diseases, including CLBP.^2,13,14^ A systematic review and meta-analysis of 13 studies involving 597 patients undergoing aquatic activities during CLBP treatment found that this form of activity reduced pain sensation, improved quality of life, and decreased physical disability.^15^

Another literature review investigated the effects of different forms of physical exercise on CLBP, and after analyzing 89 studies with 5,578 patients, the authors concluded that interventions including Pilates, stabilization, resistance, and aerobic exercises are the most effective in treating CLBP.^16^

Thus, PA is a non-pharmacological intervention often associated with lower healthcare costs.^17,18,19^ However, data on its impact on costs related to CLBP are limited. A clear understanding of the influence of PA on costs in patients with CLBP is crucial for identifying opportunities to reduce the high costs associated with CLBP in adults.

We hypothesize that regular PA can help alleviate CLBP symptoms and, consequently, reduce the demand for medical consultations and hospital admissions. Furthermore, high levels of PA may help lower public health costs.

## OBJECTIVE

This study aimed to analyze the costs of CLBP in the Brazilian National Health System and their correlates among adults after 18 months of follow-up.

## METHODS

### Sample

The Ethical Board of the Universidade Estadual Paulista (UNESP) approved the research project on April 9, 2013 (case number: 241291/2013), and the Municipal Department of Health authorized contact with the participants and the use of two facilities responsible for medical services in the city of Presidente Prudente (having approximately 200,000 inhabitants), western São Paulo State. Health units linked to the Brazilian National Health Service (NHS; in Portuguese, SUS) were chosen for convenience, and all patients signed a written consent form.

**Figure 1:**
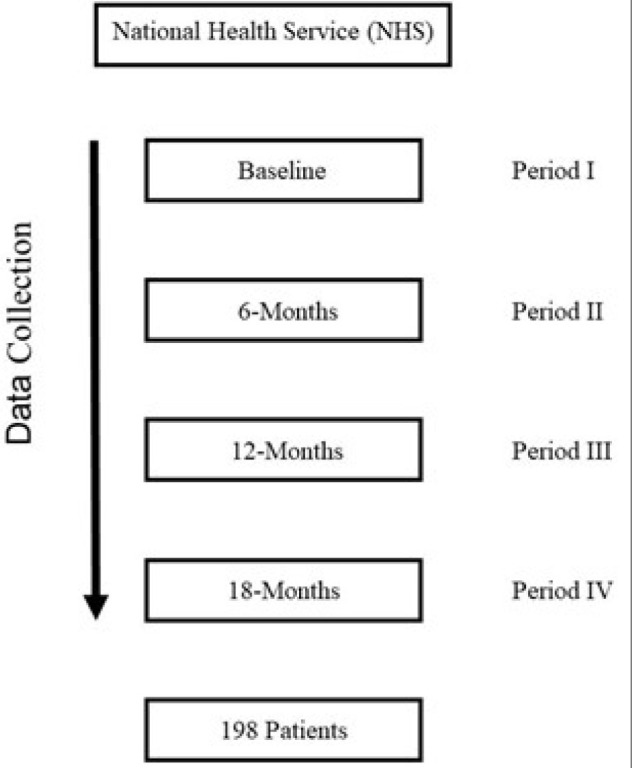
Flowchart demonstrating the collection periods used in the study.

The researchers remained in facilities linked to the Brazilian NHS for 30 consecutive days, and all patients with medical appointments who fulfilled all inclusion criteria were invited to participate in the study. The participants were contacted at four time points: baseline, 6 months, 12 months, and 18 months. Only participants with no missing data during the follow-up period were included (final sample after 18 months: 198 patients). At baseline, the inclusion criteria were active registration in the NHS, age ≥ 50 years, and residence in the metropolitan region of Presidente Prudente for at least 2 years (Figure 1).

### Chronic low back pain

The questionnaire developed by Kuorinka et al.,^20^ which was previously validated in Portuguese,^21,22^ was used to evaluate the occurrence of musculoskeletal symptoms (pain, formication, or numbness) in different regions of the body (neck, shoulder, upper back, elbows, wrists/hands, lower back, hip/thigh, knees, and ankles/feet). For each body region, there were four dichotomous questions (yes or no) related to (i) the presence of musculoskeletal disorders in the previous 12 months, (ii) impairment of daily activities in the previous 12 months due to these disorders, (iii) any health professional consultations due to these disorders, and (iv) experience with these disorders in the week immediately before the interview.

In the current study, we only considered the lower back region, and the presence of CLBP was considered positive for participants who answered “yes” to all four questions. Considering the first measurement (baseline) and the last measurement of the follow-up period (18 months), the participants were classified according to the occurrence of CLBP: none (no CLBP), yes once (presence of CLBP at either baseline or follow-up), and yes twice (presence of CLBP at both baseline and follow-up).

### Healthcare costs

The healthcare costs of each patient during follow-up were verified by the demand for the services recorded in the medical records.^19,23^ The analysis comprised all 18 months of follow-up. The following information was obtained: medicines supplied to the patient, laboratory tests performed, and the number of consultations. To convert the procedures into currency, data from the medical records were recorded, and the amounts paid by the Municipal Health Department were used. Initially, the amount of money was estimated in the Brazilian currency (Real, R$) and then converted into US Dollars (US$). Healthcare costs were classified into quartiles, with the highest quartile (≥ P75) adopted as an indicator of high healthcare costs.^19,23^

### Habitual physical activity

PA during leisure time and work was evaluated using the questionnaire developed by Baecke et al.^24^ and translated in to Portuguese by Florindo et al.^25^ The questionnaire provides a general PA score representing the sum of occupational, leisure time, and active transportation activities. Moreover, the last section of the questionnaire provides the frequency (never, rarely, sometimes, often, and always) of certain behaviors (walking, cycling, and watching television) during leisure time. In the present study, PA was identified using the general PA score and walking, cycling, and watching television behaviors.

### Covariates

Sex, chronological age, and ethnicity (1 = White, 2 = Black, and 3 = Other) were used as covariates. Economic conditions (EC) were estimated using a standard questionnaire (face-to-face interview), which divided EC into five categories, from A (highest) to E (lowest). The variables were categorized as high EC (categories A and B) and low EC (categories C, D, and E), as adopted by Fernandes et al.^13^ Finally, body weight (kg) and height (m) were collected to calculate the body mass index (BMI [kg/m^2^]). Overweight individuals (BMI ≥ 25 kg/m^2^) were identified in the sample.

### Statistical analysis

Descriptive data were presented as means, standard deviations, and 95% confidence intervals. Due to the non-parametric distribution, the Mann–Whitney U test was used to compare numerical data between the two groups. Analysis of variance and the Kruskal–Wallis test were used to compare numerical data among three or more groups when the dataset was normally and non-parametrically distributed, respectively. Spearman’s rank-order correlation (*rho*) was used to analyze the relations between the variables. Categorical variables are expressed as absolute and percentage values, with univariate statistical tests applied, including the chi-squared test (χ^2^). Significant associations were included in multivariate models (binary logistic regression) to express the magnitude of the associations in terms of the odds ratio and 95% confidence interval. Multivariate models were adjusted for covariates (sex, ethnicity, economic status, BMI, and age). Statistical analyses were performed using a specific software (BioEstat version 5.0), and the significance level adopted was 5% for all procedures.

## RESULTS

The total sample comprised 198 adults, mostly women (70.7%) and people classified as White (70.7%). The prevalence of overweight and obesity was high (78.3%) (Table 1). After the follow-up period, the prevalence of CLBP was higher in women (15%) than in men (3.4%), and younger individuals reported a higher occurrence of the outcome (Table 2).

**Table 1:** Numerical characteristics of patients aged 50 years or older who are users of two basic health units of the Brazilian health system in Presidente Prudente (SP)

Variables	Entire sample (n = 198)		Chronic low back pain			ANOVA P value
	Mean	(95% CI)	No (n = 134) Mean (SD)	Yes once (n = 41) Mean (SD)	Yes both (n = 23) Mean (SD)	
Numerical						
Age _years_	61.6	(60.4 to 62.8)	62.8 (8.6)	60.8 (8.2)^a^	55.8 (7.8)^a,b^	**0.001**
Height _cm_	157.2	(156.0 to 158.4)	157.6 (8.8)	157.2 (8.1)	154.6 (7.4)	0.298
Weight _kg_	73.0	(70.9 to 75.0)	72.6 (14)	75.5 (16.8)	70.6 (13.2)	0.379
BMI _kg/m_^2^	29.51	(28.7 to 30.2)	29.2 (5)	30.5 (6.4)	29.4 (4.8)	0.374
PA score	27.3	(26.8 to 27.9)	27.4 (4.1)	26.8 (3.5)	27.4 (3.9)	0.673
**Health care costs**	**Mean**	**(95% CI)**	**Median (IR)**	**Median (IR)**	**Median (IR)**	**Kruskal–Wallis**
Consultation	38.34	(35.40 to 41.25)	34.25 (23.21)	39.81 (26.78)	37.53 (28.25)	0.076
Exams	12.18	(9.56 to 14.81)	0 (23.99)	0 (16.46)	0 (30.94)	0.462
Medicines	46.84	(39.50 to 54.184)	27.48 (45.63)	35.88 (41.39)	46.28 (60.47)	0.210
Overall	118.65	(108.34 to 128.93)	103.69 (77.3)	114.65 (59.14)	134.26 (104.96)	0.194

Descriptive data are presented as means, standard deviations (SD), and 95% confidence intervals (CI). Analysis of variance (ANOVA) was used for comparisons between groups, with a significance level of 5% (P ≤ 0.05). BMI = body mass index; PA = physical activity; IR = interquartile range.

**Table 2:** Categorical characteristics of patients aged 50 years or older who are users of two basic health units of the Brazilian health system in Presidente Prudente (SP)

Variables			Chronical low back pain*			χ^2^ P value
	Entire sample (n = 198)		No (n = 134)	Yes once (n = 41)	Yes both (n = 23)	
Categorical	N	(%)	n (%)	n (%)	n (%)	
Sex						**0.005**
Male	58	(29,3)	47 (81)	9 (15.5)	2 (3.4)	
Female	140	(70.7)	87 (62.1)	32 (22.9)	21 (15)	
Ethnicity						0.881
White	140	(70.7)	95 (67.9)	27 (19.2)	18(12.9)	
Black	35	(17.7)	24 (68.6)	8 (22.9)	3 (8.6)	
Others	23	(11.6)	15 (65.2)	6 (26.1)	29 (8.7)	
Age						**0.006**
< 65 years	128	(64.6)	78 (60.9)	31 (24.2)	19 (14.9)	
≥ 65 years	70	(35.4)	56 (80.0)	10 (14.3)	4 (5.7)	
BMI						0.991
Normal	43	(21.7)	29 (67.4)	9 (20.9)	5 (11.7)	
Overweight/ Obesity	155	(78.3)	105 (67.7)	32 (20.6)	18 (11.7)	

Frequency analysis and the chi-squared test (χ^2^) were used to analyze the association between groups, with a significance level of 5% (P ≤ 0.05).

* Chronic low back pain was identified by four affirmative answers to the questionnaire. BMI = body mass index.

Individuals who reported any episode of CLBP had high consultation costs (with CLBP: US$ 39.62 [27.25]; without CLBP: US$ 34.25 [23.21]) (P = 0.049) (Table 3).

**Table 3:** Correlates of chronic low back pain and direct healthcare costs in patients aged 50 years or older who are users of two basic health units of the Brazilian health system in Presidente Prudente (SP)

Health care costs (US$)	Chronic low back pain*		Mann–Whitney P value
	No (n = 134)	Yes (n = 64)	
	Median (IR)	Median (IR)	
Consultations	34.25 (23.21)	39.62 (27.25)	**0.049**
Exams	0.00 (23.96)	0.00 (19.09)	0.839
Medicines	27.48 (45.65)	38.35 (47.53)	0.118
Overall	103.69 (77.40)	128.41 (75.18)	0.071

The Mann–Whitney U test was used for comparisons between groups, with a significance level of 5% (P ≤ 0.05).

*Chronic low back pain was defined as any episode of chronic low back pain during follow-up (four affirmative answers to the questionnaire). IR = interquartile range.

Comparisons between different domains of PA and healthcare costs in patients with and without CLBP identified an inverse relation between cycling and overall cost in people with CLBP (rho = -0.289, P = 0.021) but not in those without the outcome (Table 4). Moreover, a significant relation was observed between cycling and the cost of medical consultations in patients with CLBP (rho = -0.239, P = 0.021).

**Table 4:** Correlates between different domains of physical activity, presence or absence of chronic low back pain, and cost indicators in patients aged 50 years or older who are users of two basic health units of the Brazilian health system in Presidente Prudente (SP)

PA	Medical consultations		Exams		Medicines		Overall	
	rho	P value	rho	P value	rho	P value	rho	P value
Entire Sample (n= 198)								
Television	-0.001	0.987	0.072	0.314	-0.004	0.950	-0.004	0.950
Walking	-0.072	0.314	0.041	0.564	-0.075	0.291	-0.089	0.215
Cycling	-0.064	0.368	-0.099	0.167	-0.132	0.064	**-0.164**	**0.021**
Overall PA	-0.010	0.886	-0.011	0.882	-0,016	0.819	-0.052	0.465
No CLBP (n = 134)								
Television	-0.064	0.462	0.106	0.224	-0.059	0.495	-0.049	0.576
Walking	-0.112	0.196	0.058	0.507	-0.099	0.257	-0.117	0.179
Cycling	0.012	0.892	-0.079	0.365	-0.108	0.216	-0.109	0.209
Overall PA	0.034	0.701	0.064	0.461	-0.028	0.746	-0.029	0.738
Yes CLBP (n = 64)								
Television	0.100	0.433	0.005	0.971	0.129	0.311	0.085	0.506
Walking	0.095	0.457	-0.020	0.873	0.021	0.867	0.062	0.625
Cycling	-0.239	0.057	-0.193	0.127	-0.171	0.177	**-0.289**	**0.021**
Overall PA	-0.086	0.500	-0.190	0.133	0.014	0.910	-0.106	0.405

Spearman’s rank-order correlation (rho) was used, with a significance level of 5% (P ≤ 0.05); PA = physical activity; CLBP = chronic low back pain.

Finally, there was an association between more medical consultations and CLBP; however, this association was not significant after adjusting for confounders (P ≥ 0.05) (Table 5).

**Table 5:** Association and adjusted odds ratios (ORs) for chronic low back pain in patients aged 50 years or older who are users of two basic health units of the Brazilian health system in Presidente Prudente (SP)

	Consultations				Exams		Medicines		Overall	
	≥ P75			≥ P75	χ^2^	≥ P75	χ^2^	≥ P75	χ^2^
	χ^2^ p-value	OR (95% CI)	OR _adj_ (95% CI)	n (%)	P value	n (%)	P value	n (%)	P value
	n (%)								
CLBP*	0.025				0.900		0.194		0.410
No	27 (20.1)	1.00	1.00	35 (26.1)		31 (23.1)		32 (23.9)	
Yes once	13 (31.7)	**1.84 (1.00–4.02)**	1.76 (0.89–3.93)	7 (17)		9 (22)		9 (22)	
Yes both times	9 (39.1)	**2.54 (1.05–6.50)**	2.29 (0.95–6.08)	7 (30.4)		9 (39.1)		8 (34.8)	

Chi-squared test (χ^2^) was used. ORs are adjusted for sex, age, ethnicity, economic condition, body mass index, and physical activity. 95% confidence intervals (CIs) are provided. Hosmer–Lemeshow test result: P = 0.847. *Chronic low back pain (CLBP) was defined as four affirmative answers to the questionnaire. ≥ P75 = ≥ percentile 75.

## DISCUSSION

This 18-month longitudinal study found elevated occurrences of CLBP among participants, which were linked to higher healthcare expenditures associated with medical consultations, whereas cycling appeared to mitigate this relation. This analysis verified that the sample consisted mainly of women (≥ 70%) and that the prevalence of overweight and obesity was present in approximately 80% of the population. Furthermore, a prevalence of CLBP of 18.4% was found in the analyzed sample, and individuals with CLBP cost the public health system US$ 5.37 more than those without CLBP.

The greater number of women in the sample is not surprising. Women more often use the primary care services provided by the Brazilian NHS because cultural barriers decrease the number of men accessing these services.^26^ Moreover, the high number of people with a low income in the sample is justified by the fact that although the Brazilian NHS offers healthcare services to the entire population, those with lower incomes are the main users.^27^ Therefore, even though the sampling process was not representative of the entire city, some characteristics of the sample were similar to the national setting, reinforcing the idea that the selection bias in our sample was not large.

Low back pain seems to be a relevant public health problem in Brazil. In the present study, a prevalence of 18.4% was observed in the analyzed sample. These findings demonstrate values greater than those of previous studies conducted in the country. A study that investigated the occurrence of CLBP in Presidente Prudente (SP) observed a prevalence of 11.3%, whereas another study conducted in the city of Salvador (BA) found a prevalence of 14.7% in the sample analyzed.^3,28^

Studies investigating the determinants of CLBP found that higher age, female sex, and overweight/obesity were significant correlates of this outcome.^3,28^ Similar to these previous reports, sex and age were significantly associated with any episode of low back pain during our follow-up, but not overweight/obesity. The absence of significant associations may be attributed to the widespread occurrence of overweight/obesity in the analyzed sample (≥ 75%).

CLBP is a musculoskeletal outcome with the potential to increase healthcare costs.^6^ However, this phenomenon has not been thoroughly investigated in developing settings. In the USA, lower back pain is the second most common cause of medical consultations, similar to our findings, in which people with a self-report of CLBP presented costs associated with medical consultations 15.6% higher than those without a self-report of CLBP.^6,7,8^

In other countries, the burden of CLBP on individual costs is higher than that observed in the current study: € 3,100 per year in Sweden^29^ and € 1,322 per year in Germany.^30^ These differences can be explained by methodological issues, as those studies were carried out with the general population, whereas our data are exclusive to individuals older than 50 years and attended by the NHS.

Several studies have reported treatments that are beneficial for or prevent CLBP.^2,14,31^ Carvalho et al.^32^ indicated joint stabilization and control exercises to treat CLBP, while increasing PA levels has been recommended by health agencies.^33^ However, there are still doubts about the most effective exercise protocol.^34,35^ In the current study, cycling was associated with lower overall costs after 18 months only in people with self-reported low back pain. In fact, higher habitual PA (not exercise protocols) seems to mitigate healthcare costs,^19^ and this is the first report on this effect in people experiencing episodes of low back pain. Apparently, actions to increase habitual PA can be beneficial for decreasing costs in the NHS through two pathways: i) preventing the development of low back pain and ii) decreasing the costs of treatment in people suffering from low back pain.

The results of the present study can serve as an indication of the prevalence of low back pain in the Brazilian population, especially among residents in medium-sized municipalities in the state of São Paulo, and thus contribute to the implementation of public policies to minimize its occurrence. Furthermore, it is clear that the habitual practice of PA can reduce the occurrence of CLBP and the demand for health facilities by individuals who suffer from this condition, as well as reduce medical appointments and spending on medicines.

Therefore, the present study contributes to the literature by investigating the association between the practice of physical activities and a reduction in healthcare costs in patients treated by the Brazilian NHS. However, some limitations must be acknowledged. Clinical assessments to diagnose lower back pain are better than face-to-face interviews, reducing the risk of false-positive cases. Moreover, objectively measuring PA could improve the potential for identifying the burden of different PA intensities on low back pain. In addition, a national survey on this issue would improve the inferences of the findings of our study. Finally, the age of the population needs to be considered, as the population investigated was over 50 years old.

## CONCLUSION

It is possible to conclude that lower back pain is associated with greater healthcare costs, while cycling seems to be a relevant behavior that can reduce the costs for people suffering from lower back pain.
